# *Deep orange* gene editing triggers temperature-sensitive lethal phenotypes in *Ceratitis capitata*

**DOI:** 10.1186/s12896-024-00832-x

**Published:** 2024-02-01

**Authors:** Germano Sollazzo, Katerina Nikolouli, Georgia Gouvi, Roswitha A. Aumann, Marc F. Schetelig, Kostas Bourtzis

**Affiliations:** 1 Insect Pest Control Laboratory, Joint FAO/IAEA Centre of Nuclear Techniques in Food and Agriculture, Friedensstrasse 1, Seibersdorf, 2444 Austria; 2 Institute for Insect Biotechnology, Department of Insect Biotechnology in Plant Protection, Justus-Liebig-University Gießen, Winchesterstr. 2, Gießen, 35394 Germany; 3 Laboratory of Systems Microbiology and Applied Genomics, Department of Sustainable Agriculture, University of Patras, 2 G. Seferi St., Agrinio, 30100 Greece; 4 Present address: Department of Life Sciences, Imperial College London, Sir Alexander Fleming Building, South Kensington Campus, Imperial College Road, London, SW7 2AZ UK

**Keywords:** Mediterranean fruit fly, Sterile insect technique, White pupae, Tephritidae, Temperature sensitivity

## Abstract

**Background:**

The Mediterranean fruit fly, *Ceratitis capitata*, is a significant agricultural pest managed through area-wide integrated pest management (AW-IPM) including a sterile insect technique (SIT) component. Male-only releases increase the efficiency and cost-effectiveness of SIT programs, which can be achieved through the development of genetic sexing strains (GSS). The most successful GSS developed to date is the *C. capitata* VIENNA 8 GSS, constructed using classical genetic approaches and an irradiation-induced translocation with two selectable markers: the *white pupae* (*wp*) and *temperature-sensitive lethal* (*tsl*) genes. However, currently used methods for selecting suitable markers and inducing translocations are stochastic and non-specific, resulting in a laborious and time-consuming process. Recent efforts have focused on identifying the gene(s) and the causal mutation(s) for suitable phenotypes, such as wp and tsl, which could be used as selectable markers for developing a generic approach for constructing GSS. The *wp* gene was recently identified, and efforts have been initiated to identify the *tsl* gene. This study investigates *Ceratitis capitata deep orange* (*Ccdor*) as a *tsl* candidate gene and its potential to induce tsl phenotypes.

**Results:**

An integrated approach based on cytogenetics, genomics, bioinformatics, and gene editing was used to characterize the *Ccdor*. Its location was confirmed on the right arm of chromosome 5 in the putative *tsl* genomic region. Knock-out of *Ccdor* using CRISPR/Cas9-NHEJ and targeting the fourth exon resulted in lethality at mid- and late-pupal stage, while the successful application of CRISPR HDR introducing a point mutation on the sixth exon resulted in the establishment of the desired strain and two additional strains (*dor 12del* and *dor 51dup*), all of them expressing tsl phenotypes and presenting no (or minimal) fitness cost when reared at 25 °C. One of the strains exhibited complete lethality when embryos were exposed at 36 °C.

**Conclusions:**

Gene editing of the *deep orange* gene in *Ceratitis capitata* resulted in the establishment of temperature-sensitive lethal mutant strains. The induced mutations did not significantly affect the rearing efficiency of the strains. As *deep orange* is a highly conserved gene, these data suggest that it can be considered a target for the development of *tsl* mutations which could potentially be used to develop novel genetic sexing strains in insect pests and disease vectors.

**Supplementary Information:**

The online version contains supplementary material available at 10.1186/s12896-024-00832-x.

## Background

The Mediterranean fruit fly, *Ceratitis capitata* (Wiedemann), is one of the most important agricultural pests due to the damage it causes to many plant species of agronomic importance [1, 2]. The sterile insect technique (SIT), as part of area-wide integrated pest management (AW-IPM) programs, is a control tactic that has been developed to suppress, contain, and prevent the (re)introduction or locally eradicate populations of insect pests of agricultural, veterinary and human health importance [3–6].

One of the most critical aspects of SIT applications concerns the development of genetic sexing strains (GSS), which enable the mass production and separation of males and females. Male-only releases significantly improve the effectiveness and cost-efficiency of SIT applications [7–10]. Several GSSs have been developed using irradiation and classical genetic approaches for SIT applications against *C. capitata*. The two strains used nowadays are VIENNA 7 and VIENNA 8 [10, 11].

The successful development and application of these GSS depend on the presence of (i) two selectable phenotypes, the *white pupae (wp)* gene and *temperature-sensitive lethal* (*tsl*), both being located on the right arm of chromosome 5, and (ii) a Y-autosome translocation, T(Y;A), which is required to link the wild-type alleles of these genes to the male sex chromosome [10]. Females of these GSS are homozygous for the recessive alleles, sensitive to high temperatures, and emerge from white puparia, while males are heterozygous at both loci and, since they carry a single copy of the wild-type alleles for both *wp* and *tsl* loci, they are resistant to high temperatures and emerge from brown puparia [8, 10, 11].

The development of these GSS was a rather lengthy process of over two decades, entirely based on the serendipitous discovery of the *wp* and *tsl* mutations and the stochastic induction of suitable translocations (T[Y;A]). The same approach was followed for all GSS constructed using classical genetic approaches [10]. Given recent advances in the field of genome editing, a generic (neoclassical) approach was proposed for the construction of non-transgenic GSS for SIT applications [12, 13]. This approach requires the identification of gene(s) and the causal mutation(s) of suitable phenotypes, which could be used as selectable markers. The next step includes the induction of similar mutations in the orthologous gene(s) of SIT target species and the linkage of the wild-type allele of the gene marker(s) to the male sex using genome editing approaches [12, 13].

As the *wp* and *tsl* genes could be useful selectable markers for developing GSS in different SIT target species, initial efforts focused on identifying the genes responsible for these two phenotypes. The *wp* gene was recently mapped by *in-situ* hybridization to position 76B of the salivary gland polytene chromosomes and in earlier studies by deletion mapping to position 59B of the *C. capitata* trichogen polytene chromosome map [14, 15]. Based on similar transposition and deletion mapping experiments, the *tsl* gene was cytogenetically mapped at position 59B-61C [14, 16]. Based on these findings, efforts were initiated to identify the gene responsible for the tsl phenotype in *C. capitata* so that it could be used as a marker for the development of GSS in other SIT target species [12, 13]. As a first step, a tsl test (TSLT) was applied to several wild-type, GSS, and *tsl* mutant strains, and the results indicated that the lethality rates observed as a response to increasing temperatures depend on genetic and environmental factors [17]. This analysis also contributed to the identification of potential reference strains that could be used in functional tests of candidate genes [17].

*C. capitata* wild-type, GSS, and *tsl* mutant strains were recently used in genomic, transcriptomic, bioinformatic, and cytogenetic analyses to identify candidate genes in the so-called *C. capitata tsl* genomic region that may be involved in the tsl phenotype [18]. This region is defined by the *wp* gene at its left border and the *glucose-6-phosphate 1-dehydrogenase* gene (also known as *Zw*) at its right border, located at position 79C of the polytene chromosome map [18, 19]. It is 6,200,460 bp long and contains 561 genes [18]. The results of this integrated and comparative approach led to the identification of 33 *Drosophila melanogaster* temperature sensitive genes with orthologs in the *C. capitata tsl* genomic region. In addition, 214 polymorphisms were detected in 19 out of the 33 genes including locus LOC101455833 (*vacuolar protein sorting-associated protein 18 homolog, VPS18)* also known as the *deep orange* gene in *D. melanogaster* (*Dmdor*) (Gene ID: CG3093) [18].

The *deep orange* gene plays a major role in vesicle-mediated protein trafficking to lysosomal compartments and in membrane docking/fusion reactions of late endosomes/lysosomes probably as part of the class C core vacuole/endosome tethering (CORVET) complex, as previously reported [20–23]. It is essential in larval neuromuscular junctions for endosomal sorting and trafficking old or dysfunctional synaptic vesicle proteins through a degradative endolysosomal route [22]. Moreover, it is essential for the biogenesis of eye pigment granules [21] and for maintaining normal levels of the protein Rush hour, which functions in endosome formation and trafficking [24].

Several *Dmdor* mutations have been associated with lethal phenotypes appearing at the third instar larval, pre-pupal, or pupal stage, with some mutations being temperature sensitive [19, 24, 25]. Exposure to elevated temperatures may not only result in lethality, but might also affect eyes, wings, late endosomes, thorax, and macrochaeta [20, 26].

The *C. capitata* orthologue of the *deep orange* gene (hereafter *Ccdor*) was selected as a potential *tsl* candidate gene and was targeted via Clustered Regularly Interspaced Short Palindromic Repeats (CRISPR/Cas9) gene editing for further characterization. It is known that CRISPR/Cas9 genome editing can be used to target specific genes introducing a double-strand break (DSB), which can be repaired in two ways: by the non-homologous end-joining (NHEJ) or the homology-directed repair (HDR) [27, 28]. Both pathways can be exploited for gene editing. Performing NHEJ, mutations are induced, and the repair system implies the introduction of random INDELs (insertions or deletions), mainly used to knock out genes. Carrying out HDR, a DNA donor template is used to modify a specific region [29]. CRISPR/Cas9 has been successfully applied in many insect pest species targeted by SIT, such as *Ceratitis capitata* [15, 27, 28, 30], *Bactrocera tryoni* [15, 31, 32], *Bactrocera dorsalis* [33, 34], *Anastrepha suspensa* [35] and *Zeugodacus cucurbitae* [36].

In the present study, we investigated whether the *C. capitata dor* gene is involved in a temperature-lethal phenotype in this species. We used CRISPR/Cas9-mediated NHEJ to knock out the *Ccdor* gene targeting the fourth exon and CRISPR/Cas9-mediated HDR to introduce a specific point mutation in the sixth exon and characterized the mutant strains with an emphasis on the expression of temperature-sensitive lethal phenotypes.

## Results

### Ceratitis capitata deep orange gene

**Fig. 1:**
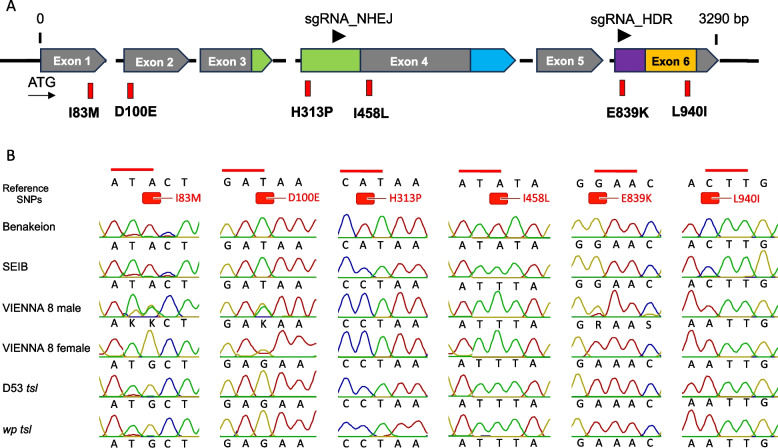
**A** Schematic representation of the *Ccdor* gene spanning six exons. *Pep3/Vps18/deep orange family* domain, Clathrin/VPS domain, Helo_like_N domain, Ring finger/U-box domain, and sgRNAs are shown in green, blue, purple, orange, and black, respectively. Red dashes represent the six SNPs that lead to amino acid changes identified via wild type and *wp tsl* mutant strains' *deep orange* genomic sequence comparison. **B** *Ccdor* gene SNPs Sanger sequenced in Benakeion Volos FF26 (hence Benakeion) (wild-type), Seibersdorf FF26 (hence SEIB) (wild-type), VIENNA 8 2010 FF26 (hence VIENNA 8) (GSS), D53-3–28 FF21 (hence D53 *tsl*) (mutant) and *wp tsl* FF21 (hence *wp tsl)* (mutant) strains using as reference *Ccap 3.2.1* (accession GCA_905071925.1) genome

The *Drosophila melanogaster deep orange* gene orthologue in *C. capitata (Ccdor)* is characterized by a length of 3,290 bp (973 aa) (RNAseq and genomic data – NCBI BioProject No PRJEB57574), a total of 6 exons (Fig. 1A), and 56.14% identity at the amino acid level with its *D. melanogaster* orthologue. Combined results from the NCBI Conserved Domain Database Server and SMART predicted the presence of four conserved domains for *Ccdor*: a *Pep3/Vps18/deep orange family* domain (300–452 aa), a Clathrin/VPS domain (627–772 aa), a Helo_like_N domain (804–865 aa) and a Ring finger/U-box domain (861–948 aa) (Fig. 1A). The *C. capitata deep orange* gene is localized on the right arm of chromosome 5, in position 77B of the polytene chromosome map, as shown by *in-situ* hybridization analysis (Figure S1).

As reported in our previous study, polymorphism calling using the VIENNA 7 GSS (female) and *wp tsl* mutant strain (male and female) Illumina NGS data (NCBI BioProject No PRJEB57574) identified 36 SNPs in the respective *Ccdor* gene coding sequences [18]. The present study confirmed them via Sanger sequencing in the *wp tsl* strain. Six of them lead to amino acid changes (I83M, D100E, H313P, I458L, E839K, L940I). H313P is in the *Pep3/Vps18/deep orange family* domain, E839K in the Helo_like_N domain, and L940I in the Ring finger/U-box domain (Fig. 1A; Table S1). These six positions were checked in several wild-type, GSS, and *tsl* mutant strains (Table 1) to confirm their homozygosity in wild-type and *tsl* mutant strains (including GSS females) and their heterozygosity in GSS males, a pattern which should be expected for a *tsl* mutation (Fig. 1B). One of them (E839K) followed the proper pattern in all studied strains (Fig. 1B). Further analyses were conducted to assess the conservation of DOR protein and the conservation of these positions across various species to evaluate *dor*’s eligibility as a selectable tsl marker (Figure S2). This assisted in identifying and prioritizing suitable target sites for CRISPR/Cas9 genome editing. The findings revealed that the DOR protein has a pairwise identity of 59.3% among insect species, which increases to 91.3% when only Tephritids are considered (Figure S2). Moreover, its secondary structure was evaluated and predicted by Phyre^2^ and it contains 42% alpha helix, 19% beta-strand, and 22% disordered regions (Figure S3). Additionally, it was observed that three out of the six amino acids, in which polymorphisms were identified in *tsl* mutant strains, exhibit high conservation among insect species (Figure S2) and are located in the alpha helix (E839K & L940I) and disordered regions (D100E).

**Table 1:** *Ceratitis capitata* strains used in the present study

	**Strain**	**Group**	**Used for**
1	Egypt II FF26	wild type	Illumina NGS, 10X Genomics, *tsl* test
2	Benakeion Volos FF26	wild type	Sanger sequencing, RNA-Seq
3	Seibersdorf FF26	wild type	Sanger sequencing
4	VIENNA 8 2010 FF26	GSS	Sanger sequencing
5	VIENNA 7	GSS	Illumina NGS, 10X Genomics
6	*wp tsl* FF21	mutant	Illumina NGS, 10X Genomics, Sanger sequencing, RNA-Seq, *tsl* test
7	D53-3–28 FF21	mutant	Sanger sequencing

### Knock-out of the *Ccdor* gene in exon 4 causes lethality at the pupal stage

The sgRNA_NHEJ (Table S2) targeting exon 4 and recombinant Cas9 protein were injected in 586 Egypt II FF26 (hence EgII) embryos to knock out the *Ccdor* gene. After injection, 145 embryos reached the larval stage, 70 reached the pupal stage, and 55 eclosed as adults (Table S3). Fifteen dead pupae were analyzed genotypically for CRISPR/Cas9-induced *dor* mutations; seven had NHEJ events (Figure S4).

**Fig. 2:**
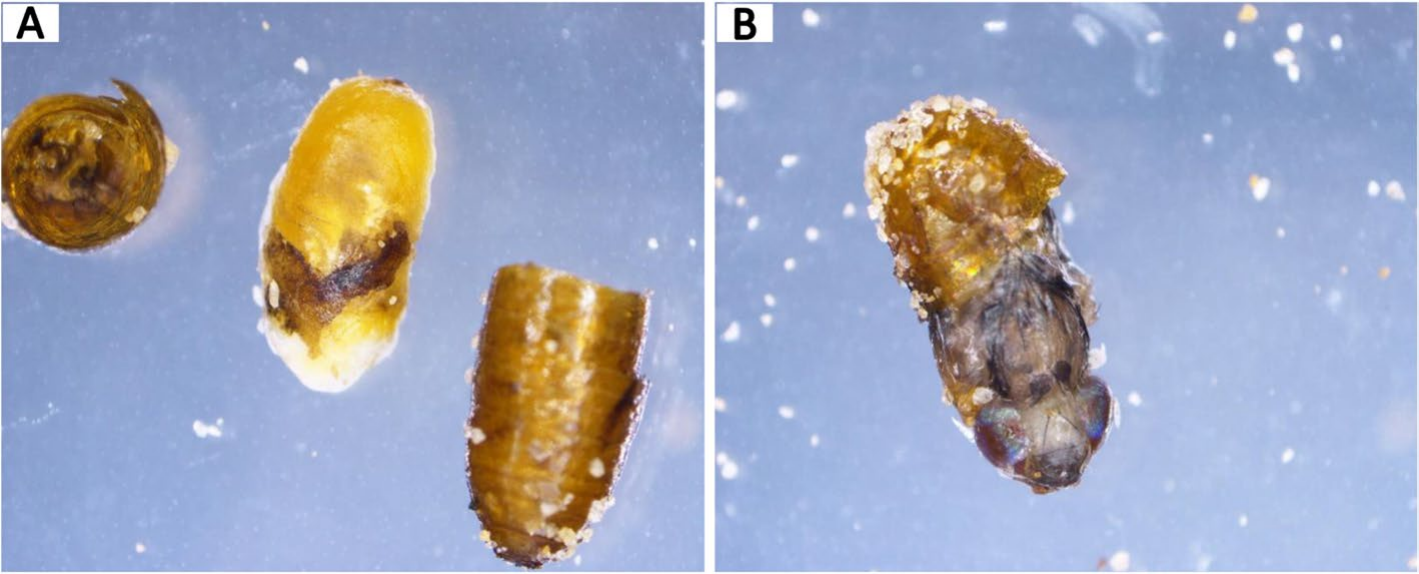
Lethal phenotype of *Ccdor* gene knock-out. Lethality was observed during mid- (**A**) and late-pupal stage (**B**) following *Ccdor* gene knock-out in exon 4 by CRISPR/Cas9 NHEJ targeted mutagenesis

Surviving adults were individually backcrossed to EgII wild-type virgin individuals and eggs from those crosses were collected three times in two-day intervals. G_1_ adults were inbred and G_2_ adults were subjected to non-lethal genotyping. 425 G_2_ adults were screened for CRISPR-induced mutations, but none were detected. This suggested that all G_0_ individuals with mosaic genotypes (Figure S4) died at mid- or late-pupal stage (Fig. 2), most likely due to the mutation that took place.

### *Deep orange* E839K mutation introduced in EgII using CRISPR/Cas9 HDR

**Fig. 3:**
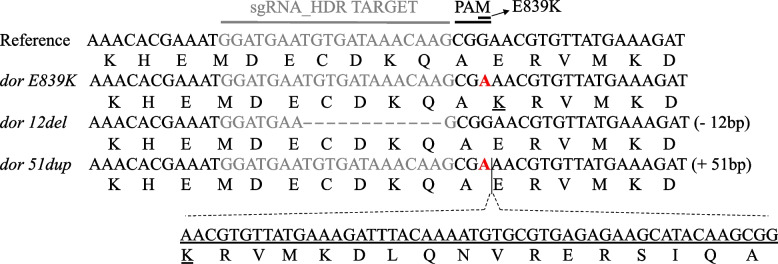
CRISPR strains obtained after targeting *Ccdor *via CRISPR HDR. The *Ccdor* reference sequence (*Ccap 3.2.1* (accession GCA_905071925.1)) with the sgRNA target sequence in grey and the PAM site is shown in the first row. The rows below represent the three mutant strains obtained via CRISPR HDR with the SNP responsible for the *E839K* mutation (*dor E839K*), the 12 bp deletion (*dor 12del)*, and the 51 bp duplication inserted via false integration of the HDR repair template (*dor 51dup)*, respectively. The nucleotide change responsible for the E839K mutation is shown in red, while the duplication is underlined

Recombinant Cas9 protein, a sgRNA targeting the sixth exon (sgRNA_HDR) of *Ccdor,* close to the E839K (G2889A) mutation and a short single-stranded repair template (151 nt), designed in sense orientation of the gene (Table S2), were injected into 255 *Cc* EgII embryos (Table S3). In addition, 100 *Cc* EgII embryos were injected using the same mix but replacing the sense-oriented single-stranded repair template with the antisense-oriented one (Table S3). Twenty-eight adults survived the injections using the sense-oriented ssODN, and three survived using the antisense-oriented ssODN. Differently from what was done during the NHEJ experiment, G_0_ adults were individually backcrossed to *wp tsl* FF21 (hence *wp tsl*) mutant strain virgin mates, trying to obtain complementation of the CRISPR allele with the *tsl* mutant one with the consequent manifestation of the desired phenotype. Using non-lethal genotyping on G_1_ adults, we determined that at least one G_0_ family, injected using the sense-oriented single-stranded repair template, produced HDR-positive offspring. Positive siblings were single-pair mated to EgII wild-type individuals of the opposite sex to remove the *wp tsl* background thus avoiding any impact on the tsl phenotype arising from its presence. Crosses, non-lethal genotyping and Sanger sequencing were performed during the next generations to isolate flies carrying the E839K mutation. Once identified, they were inbred to obtain a homozygous mutant strain (Fig. 3). No change in eye color or other visible mutant phenotype was detected.

### *Deep orange* CRISPR NHEJ events obtained during the CRISPR HDR experiments

In addition to the successful CRISPR HDR performed to introduce the E839K point mutation, the genotyping of G_1_ embryo pools suggested the presence of at least three G_0_ flies with editing events different from the expected one. Genotyping of G_1_ adults showed flies with mosaic genotypes (Figure S4). However, unlike the latter ones, induced mutations were viable. We used non-lethal genotyping to determine that at least two G_1_ families showed different NHEJ events. Crosses and non-lethal genotyping were performed during G_2_ and G_3_ to isolate the single events: a deletion of 12 bp (TGTGATAAACAA) and a duplication (which also included the E839K HDR event) of 51 bp (AAACGTGTTATGAAAGATTTACAAAATGTGCGTGAGAGAAGCATACAAGCG), both in frame (Fig. 3), presumably produced as a result of erroneous repair by the DNA polymerase. Once flies homozygous for the 12 bp deletion and the 51 bp duplication were identified, they were inbred at G_4_ to establish the respective homozygous strains (*dor 12del* and *dor 51dup*) (Fig. 3). These NHEJ events were unrelated to eye color change or other visible mutant phenotypes.

### Temperature-sensitive lethal tests of the three dor mutant strains

**Fig. 4:**
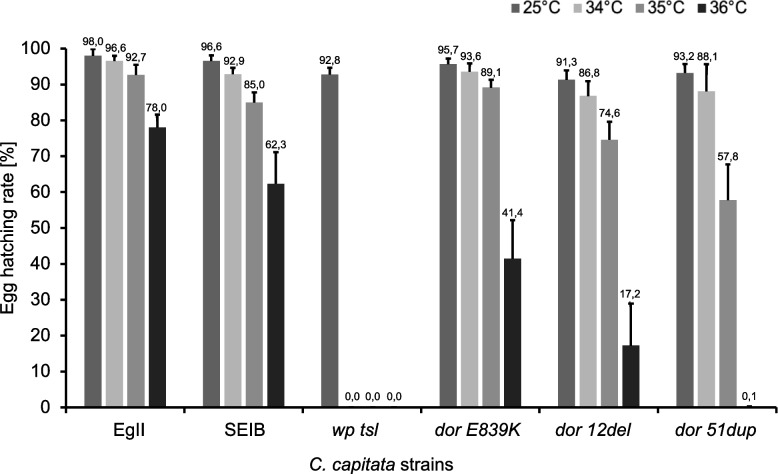
Egg hatching rates of wild-type control (EgII, SEIB), mutant (*wp tsl*), and CRISPR-mutant (*dor E839K, dor 12del,* and *dor 51dup*) strains. Egg hatching rates (shown as mean ± standard deviation) of strains reared without heat-shock treatment at 25 °C and after 24 h heat-shock treatment at 34 °C, 35 °C or 36 °C are shown. Values represent the mean of the three replicates for the three tested days

Egg hatching, pupal recovery, and adult emergence rates were assessed for all homozygous CRISPR-mutant (*dor 12del, dor 51dup,* and *dor E839K*) and control strain (wild type: EgII, SEIB, and mutant: *wp tsl*) (Table S4), based on the initial 100 embryos collected per each of the three replicates. Statistically significant differences were detected at the egg hatching rate among all the strains tested at 25 °C (F = 9.1437, df = 48, *p* = 3.674 × 10^–6^), 31 °C (F = 54.733, df = 48, *p* < 2.2 × 10^–16^), 32 °C (F = 18.745, df = 48, *p* = 2.746 × 10^–10^), 33 °C (F = 260.49, df = 48, *p* < 2.2 × 10^–16^), 34 °C (F = 686.77, df = 48, *p* < 2.2 × 10^–16^), 35 °C (F = 79.944, df = 48, *p* < 2.2 × 10^–16^) and 36 °C (F = 72.464, df = 48, *p* < 2.2 × 10^–16^) (Fig. 4, Table S4, Table S5).

At the embryonic stage, *dor 12del* and *dor 51dup* egg hatching rates at 25°C were significantly different from those observed in the wild-type strains EgII and SEIB (Table S6), indicating fitness cost, albeit minimal. On the contrary, no difference was detected among the *dor E839K* and the two wild-type strains. Thermal sensitivity of wild-type and CRISPR strains started at 35°C (Table S5), and egg hatching rates ranged between 92.67 ± 2.81% (EgII) and 57.78 ± 9.94% (*dor 51dup*) (Table S4). At 36°C, the egg hatching rate of the EgII and SEIB strains was 78.00 ± 3.59% and 62.33 ± 8.81%, respectively, while that of the three CRISPR strains was significantly decreased (*dor 12del *= 17.22 ± 11.70%*, dor 51dup *= 0.11 ± 0.19% and *dor E839K *= 41.44 ± 10.75%; Table S4). Interestingly, at 36°C, *dor 51dup* and *wp tsl* were not statistically different (Table S6), indicating a similarity between the behavior of the CRISPR strain and the original *tsl* strain.

**Fig. 5:**
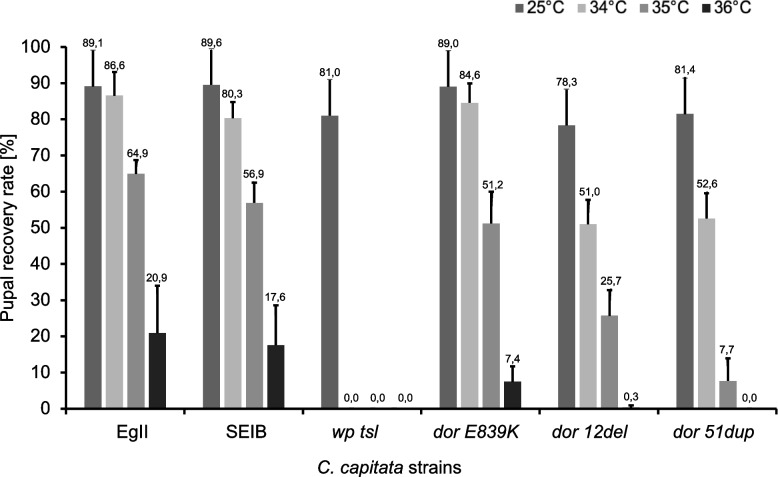
Pupal recovery rates of control (wild type: EgII, SEIB, and mutant: *wp tsl*) and CRISPR-mutant (*dor E839K, dor 12del,* and *dor 51dup*) strains. Pupal recovery rates (shown as mean ± standard deviation) of strains reared at 25 °C without heat-shock treatment and after 24 h heat-shock treatment at 34 °C, 35 °C and 36 °C are shown. Values represent the mean of the three replicates for the three tested days

Statistically significant differences were also detected for pupal recovery among all the strains tested at 25 °C (F = 2.5388, df = 48, *p* = 0.04064), 31 °C (F = 27.071, df = 48, *p* = 6.635 × 10^–13^), 32 °C (F = 18.848, df = 48, *p* = 2.524 × 10^–10^), 33 °C (F = 10.445, df = 47, *p* = 8.825 × 10^–7^), 34 °C (F = 28.269, df = 40, p = 3.475 × 10^–11^) and 35 °C (F = 29.097 df = 40, p = 2.281 × 10^–11^). At 36 °C, *wp tsl* and *dor 51dup* strains did not show any pupal recovery (Table S4) (F = 2.518, df = 28, *p* = 0.07845) (Fig. 5, Table S4, Table S7).

A significant difference was observed among the different strains concerning the temperature at which the pupal recovery rate started to decrease (Table S7). The *wp tsl* mutant strain was shown to be the most sensitive since the pupal recovery rate started to reduce at 31 °C, while for *dor 12del* and *dor 51dup,* the reduction was initiated at 33 °C, for *dor E839*K at 34 °C (Table S7), and for the wild-type strains at 35 °C, respectively (Table S7). In addition, the TSLT results provided clear evidence that the pupal recovery rate of the *dor 51dup* strain drastically decreases between 34 °C (52.56 ± 6.98%) and 35 °C (7.67 ± 6.24%) (Table S7), while for *dor 12del* (35 °C = 25.67 ± 7.10; 36 °C = 0.33 ± 0.58%) and *dor E839K* (35 °C = 51.22 ± 8.74; 36 °C = 7.44 ± 4.27%), an exposure at 36 °C was required (Table S7).

**Fig. 6:**
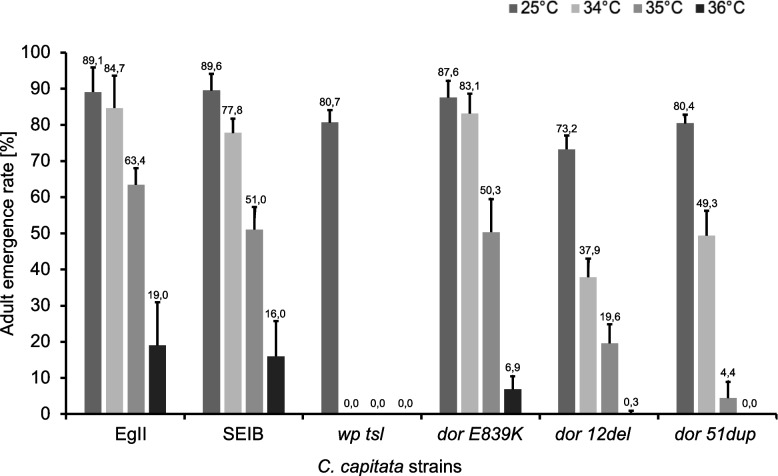
Adult recovery rates of control (wild type: EgII, SEIB, and mutant: *wp tsl*) and CRISPR-mutant (*dor E839K, dor 12del,* and *dor 51dup*) strains. Adult emergence rates (shown as mean ± standard deviation) of strains reared at 25 °C without heat-shock treatment and after 24 h heat-shock treatment at 34 °C, 35 °C and 36 °C are shown. Values represent the mean of the three replicates for the three tested days

Statistically significant differences were also detected for adult emergence among all the strains tested at 25 °C (F = 14.223, df = 48, *p* = 1.541 × 10^–8^), 31 °C (F = 112.10, df = 48, *p* < 2.2 × 10^–16^), 32 °C (F = 211.1, df = 48, *p* < 2.2 × 10^–16^), 33 °C (F = 8.7947, df = 40, *p* = 3.424 × 10^–5^), 34 °C (F = 434.56, df = 40, *p* < 2.2 × 10^–16^) and 35 °C (F = 10.909, df = 40, *p* = 4.481 × 10^–6^), while at 36 °C (F = 0.2303, df = 23, *p* = 0.7961) no statistical differences were found (Fig. 6, Table S4, Table S8).

The impact of exposure to high temperatures on adult emergence differed among CRISPR and control strains. The adult emergence rate of the *wp tsl* mutant strain decreased at 31 °C (Table S7) that of EgII and *dor 12del* at 34 °C, while *dor 51dup* at 35 °C, respectively (Tables S4 and S8). It is worth noting that when embryos were subjected to a 24-h heat treatment at 36 °C, the observed lethality was 100% for *dor 51dup* and almost 100% (99.67 ± 0.58%) for *dor 12del* (Tables S4 and S8).

## Discussion

The temperature-sensitive lethal (tsl) phenotype has been used as a selectable marker in the most successful *C. capitata* GSS, VIENNA 7 and VIENNA 8, developed so far [10, 11]. More than two billion sterile GSS males are being produced in mass-rearing facilities every week and released to control populations of this major agricultural pest worldwide. Identifying the *tsl* gene and characterizing the mutation(s) responsible for the respective phenotype will pave the way for using it as a selectable marker for developing GSS in other SIT target species [10, 12, 13, 37–39]. In the present study, we characterized the *deep orange* locus of *C. capitata*. This gene is known to have temperature-sensitive lethal mutations in *D. melanogaster*, and we investigated whether a tsl phenotype, similar to the ones reported previously, can be reproduced by inducing CRISPR/Cas9 mutations in *Ccdor* [21, 25, 40].

The *Ccdor* gene was selected as a candidate *tsl* gene by thoroughly analyzing the *tsl* region on chromosome 5 [18]. Four sets of data were pointing towards that candidate: (a) the most recent genome assembly suggested the presence of *Ccdor* on the right arm of chromosome 5. Its position was confirmed by *in-situ* hybridization on polytene chromosomes, which localized *Ccdor* in 77B, in the area where the *tsl* gene is expected to be [14, 16]; (b) mutations in its *D. melanogaster* orthologue have resulted in a tsl phenotype [21, 25]; (c) the presence of point mutations resulting in amino acid substitutions when *Ccdor* gene sequences were compared between wild type and *tsl* mutant strains [18] and (d) its highly conserved amino acid sequence among insects, making it suitable for the construction of GSSs in other SIT target species.

CRISPR/Cas9-NHEJ targeting the *Ccdor* functional domain *Pep3/Vps18/deep orange,* present in exon 4, resulted in non-viable progeny with lethality being observed at the mid- and late-pupal stages (Fig. 2A and B). This contrasts observations in *D. melanogaster*, where lethality occurred during larval and pre-pupal stages [20, 25, 26, 41, 42]. This difference in lethality stages observed in *Ccdor* after the knock-out may be influenced by the specific indel(s) introduced. However, the exact nature of the indel(s) has not been determined due to the genetic mosaicism encountered during G_0_.

CRISPR/Cas9-HDR gene editing in the EgII wild-type strain introduced a point mutation detected on the sixth exon of the *Ccdor* gene of the *tsl* mutant strain. This resulted in the desired correction and two additional mutations: a deletion and a duplication, both in frame, due to an error in the use of the repair template during the double-strand break sealing process. The efficiency of CRISPR HDR, including the appearance of errors, can be impacted by various factors, such as the activity of the endogenous repair systems, the cell cycle, and the length of the homology arms of the repair template [43]. The three strains obtained, *dor E839K*, *dor 12del*, and *dor 51dup*, are all temperature-sensitive lethal, but showed differences from the original *tsl* mutant strain. The embryonic lethality in the CRISPR *Ccdor* strains appears at higher temperatures than the original *tsl* mutant strain. Notably, only the *dor 51dup* strain exhibited almost complete embryonic lethality at 36 °C, but all three strains reached high lethality rates at an early larval stage*.* The rearing efficiency of the new strains was satisfactory, and the *dor* gene is a valuable target for developing GSS in other species. However, more investigations are needed to employ *dor* as a selectable marker in *Ceratitis capitata*, and to explore the genetic background and possible involvement of other loci in the tsl phenotype.

*Deep orange* has several domains with diverse and essential functions. As the protein plays an important role in cellular activities, mutations in these domains may lead to loss/gain of function or significantly affect cellular activities, resulting in lethality [20, 44, 45]. The *Pep3_Vps18* domain, targeted by CRISPR NHEJ in the present study, is involved in endosomal sorting and vesicle trafficking [20, 44, 45]. Knock-out mutations in this domain have caused severe defects in endosomal sorting, vesicle trafficking, multivesicular body (MVB) formation, and increased levels of cellular stress and oxidative damage in yeast and mammalian cells [46, 47]. Thus, this might also be responsible for the lethal effect we experienced in the knock-out via NHEJ targeting in this study. The function of the *Helo_like_N* domain, which was targeted via CRISPR (HDR), is not fully understood. Previously, it was reported to play a role in RNA processing, DNA binding, transcriptional regulation, and cell death-inducing activity [48–52]. Moreover, mutations within the Helo_like_N domain, particularly in its transmembrane helix region, may compromise its function [53]. As shown in Figure S3, the E839K mutation, the deletion *dor 12del*, and the duplication *dor 51dup* are all located in an α-helix region. This could affect the regular gene expression pattern, leading to cellular function and development changes, including the gain of temperature sensitivity [50]. Although temperature sensitivity (ts) has been commonly associated with point mutations, deletions and duplications/insertions have also been reported, albeit less frequently, as the causal factor of tsl [30, 32, 54–74]. Such mutations may alter a functional domain, the overall native structure, or the specific activity of the protein and these may be potential explanations for the tsl phenotype of *dor 12del* and *dor 51dup* strains [73, 74].

Mutations in the *ring finger/U-bo*x domain may replace highly conserved cysteine residues needed to form the "U-shaped" beta-sheet, destabilizing the protein and affecting its function [75–77]. This is the case of the *D. melanogaster* temperature-sensitive lethal mutation *dor*^*1*^ (C979Y) [21]*.* When insects carrying the *Dm dor*^*1*^ mutation are exposed to high temperatures, they die at the pupal stage [25] and present altered phenotypes in the eyes, thorax, and wings [20]. The *C. capitata tsl* mutant strain also has a non-synonymous point mutation in the same domain (L940I; Table S1). Whether this mutation has any effect on the tsl phenotype awaits investigation. It should be mentioned, however, that the expression of the typical tsl phenotype observed in the *C. capitata tsl* mutant strains may require the combination of point mutations in more than one domain of the *Ccdor*; for example, the E839K mutation in the *Helo_like_N* domain combined with the L940I in the *ring finger/U-bo*x domain or mutations in other domains or even other genes [78]. This was previously observed in *D. melanogaster* in genes involved in the control of body size [79], behavior [80], or tumor suppression [81]. Finally, none of the mutations in this study in the *Ccdor* resulted in eye colour alteration or any other visibly detectable phenotypes.

## Conclusions

The successful application of CRISPR/Cas9 genome editing targeting the *deep orange* gene of *Ceratitis capitata* resulted in three mutant strains that proved to be temperature sensitive. The presence of a 51 bp duplication together with the E839K mutation (*dor 51dup*) in the *Ccdor* coding region triggers total embryonic lethality following heat shock at 36 °C. In addition, for all the CRISPR strains (*dor 51dup*, *dor 12del,* and *dor E839K),* a variable lethality was observed during the larval and pupal stages following heat shock at 35 °C. Although two of the three CRISPR strains, *dor 51dup,* and *dor 12del*, exhibited minimal fitness cost at the embryonic stage when reared at 25 °C, all gene-edited strains present a high productivity rate suggesting their suitability for breeding. Given this characteristic and the high conservation of the Deep orange protein sequence among insects, particularly Tephritids, the *dor* gene emerges as a promising selectable marker for creating new genetic sexing strains (GSS).

## Methods

### *Ceratitis capitata*: strains and fly rearing.

In the frame of this study, seven *Ceratitis capitata* strains were used (Table 1) and reared under standard laboratory conditions (24 ± 2 °C, 55 ± 10% RH, and 14/10 h light/dark cycle) as previously reported in Sollazzo et al., 2022 [17].

### Analysis of *Dm deep orange* gene orthologue in *Ceratitis capitata*

Using the *Cc deep orange* protein sequence (XP_004536447.1), a search for conserved domains was carried out through the NCBI Conserved Domain Database server (http://www.ncbi.nlm.nih.gov/cdd/cdd.shtml) [82] and the SMART online tool (http://smart.embl-heidelberg.de/) [83, 84]. The detected domains were annotated on *Ccdor* genomic sequence in Geneious Prime 2022.1.1 to check if any polymorphism found in its coding sequence (CDS) [18] was located inside a conserved domain. Secondary structure and disorder predictions have been carried out using Phyre^2^ (http://www.sbg.bio.ic.ac.uk/phyre2) and the *Ccdor* wild-type protein sequence as input [85].

### DNA extraction and Sanger sequencing of the *deep orange* gene

Genomic DNA was extracted from three males and three females of *C. capitata* Benakeion, SEIB, VIENNA 8, *wp tsl*, and D53 *tsl* strains using ExtractMe DNA tissue kit (Blirt, Poland) following the manufacturer’s instructions. A NanoDrop spectrometer was used to assess the quantity and quality of the extracted DNA. Primers (Table S2) were designed using the Geneious Prime 2022.1.1 software. PCRs were performed in a 25 µL reaction volume using 12.5 μL Platinum™ Green Hot Start PCR Master Mix (2X) Kit (Thermo Fisher Scientific), 60–80 ng DNA, and the following PCR settings [94 °C, 2 min; 35 cycles of (94 °C, 30 s; 60 °C, 30 s; 72 °C, 120 s); 72 °C, 5 min]. PCR products were analyzed by electrophoresis in 2% agarose gels and visualized under UV light. Amplicons were purified using the DNA Clean & Concentrator-25 kit according to the manufacturer's protocol (Zymo Research—Irvine, CA, USA). The purified products were adjusted to the concentration of 10 ng/µl while sequencing primers were diluted following the Eurofins Genomics instructions up to 100 nmol/µl. The sequencing mix was prepared in a final volume of 15 µl (13 µl of DNA and 2 µl of primer). Sequencing results were imported in Geneious Prime 2022.1.1 and aligned to the *Ccdor* gene wild-type sequence extracted from *Ccap 3.2.1* (accession GCA_905071925.1) using the Geneious Prime “Map to reference” tool with default parameters.

### CRISPR/Cas9 genome editing

Lyophilized Cas9 protein from *Streptococcus pyogenes* (CP01—PNA Bio, Newbury Park, California, USA) was resuspended in nuclease-free water to 1 μg/μl, separated in aliquots, and stored at − 80 °C until further use. Single guide RNAs: sgRNA_NHEJ (TCAAAATGCACCACGTGCCA) and sgRNA_HDR (GGATGAATGTGATAAACAAG) were designed and checked for off-targets using the “Find CRISPR site” tool in Geneious Prime 2022.1.1 [86] using the *Ceratitis capitata* 2.1 genome (accession GCF_000347755.2) from NCBI as the off-target database. sgRNAs were ordered from Sigma Aldrich, Germany, with the following specifications (Physical material: Synthetic RNA, Purification: HPLC, CRISPR species: SpCas9, Structure: sgRNA (crRNA + tracrRNA as one), Scale of synthesis: 3 nmol, modified, dry).

The two 151 bp single-stranded donor templates for CRISPR HDR, “ssODN_E839K_sense” and “ssODN_E839K_anti”, designed in sense and antisense orientation (Table S2) to the double-strand break (DSB) [87] to re-build the mutation in position 839 (E839K), were synthesized by Eurofins Genomics (EXTREMer oligo, purified salt-free, quality control by CGE). They differ from the wild-type sequence by three bases (72A > G, 75G > C, 76G > A). The change in position 76 of the ssODN (G > A; Glu839 > Lys839) re-builds the mutation found in the *wp tsl* mutant strains while the second 75 (G > C; Ala838 > Ala838) and the third 72 (A > G; Gln837 > Gln837) are silent mutations to reduce the target sequence similarity after HDR and mutate the PAM site to prevent re-editing by the CRISPR/Cas9 machinery [88, 89].

### Embryonic microinjections for CRISPR-Cas9 targeting

The injection mix for CRISPR NHEJ and HDR contained 360 ng/μl Cas9 protein, 200 ng/μl sgRNA, and a final concentration of 300 mM KCl in a 10 μl volume, as described in previous studies [27, 90, 91]. The mix was subjected to 10 min incubation at 37 °C to complex the sgRNA and Cas9 protein. For CRISPR HDR, we added 200 ng/μl ssODN (sense or antisense) after the incubation step; also previously described [27, 28, 30, 90, 91].

Microinjections were carried out in 40–45 min old wild-type *C. capitata* EgII embryos which were previously chemically dechorionized (up to 1-day old solution of 2.8% sodium hypochlorite, 3 min), fixed on double-sided sticky tape (Scotch 3 M), dehydrated (93% calcium chloride, 6 min) and covered with halocarbon oil 700 (Sigma-Aldrich) [92]. Microinjections were performed using siliconized quartz glass needles (Q100-70–7.5; Sutter Instruments, Novato, CA USA) drawn out on a laser-based micropipette puller (Sutter P-2000) with the following conditions (Heat = heat, Filament = Fil, Velocity = Vel, Delay = Del, Pull = Pull): Quartz (Q100-70–7.5): Heat 750, Fil 5, Vel 70, Del 130, Pull 175, a FemtoJet 4X micromanipulator/microinjector (Eppendorf, Hamburg, Germany) and a Leica DM IL LED inverted microscope (Leica Microsystems, Wetzlar, Germany). Once injected, embryos were kept at 25 °C and 60% RH until larval hatching and transferred from the oil to the larval food using a brush.

### Molecular detection of CRISPR/Cas9-induced *deep orange* mutations

Genomic DNA extraction from single G_0_ flies and pupae, PCRs, DNA purification, and Sanger sequencing were performed as described above in “DNA extraction and Sanger sequencing of *deep orange* in *Ceratitis capitata* strains”. G_2_ and G_3_ flies were analyzed via non-lethal genotyping using an adapted version of the protocol established by Carvalho et al. [93], using a single adult leg and Platinum™ Direct PCR Universal Master Mix kit (Thermo Fisher Scientific). In more detail, PCRs were performed by cutting single legs from anesthetized flies using micro scissor (Hammacher Karl, Germany), placing each one of them into single PCR tubes containing 12.5 μL Platinum™ Direct PCR Universal Master Mix (Thermo Fisher Scientific), primers (100 nM) and water up to a final volume of 25 μL. The following pairs of primers were used: P58_NHEJ_geno_F / P58_NHEJ_geno_R for NHEJ and P58_HDR_geno_F / P58_HDR_geno_R for HDR, respectively (Table S2). PCR settings were the following: [94 °C, 2 min; 35 cycles of (94 °C, 15 s; 60 °C, 15 s; 68 °C, 20 s); 68 °C, 5 min]. The 940 bp (CRISPR NHEJ) and the 829 bp (CRISPR HDR) PCR products were then verified by gel electrophoresis, purified using the DNA Clean & Concentrator-25 kit according to the manufacturer's protocol (Zymo Research—Irvine, CA, USA), Sanger-sequenced (NHEJ: P58_NHEJ_geno_F; HDR: P58_HDR_geno_F) using Eurofins Genomics Tube service and analyzed in Geneious Prime 2022.1.1.

### Crossing and screening

G_0_ adults that survived to sexual maturity were individually crossed to three EgII wild-type (CRISPR NHEJ) or *wp tsl* (CRISPR HDR) virgin mates (Table S3). Eggs were collected three times, and G_1_ adults were inter-crossed in mass, resulting in three potential G_2_ genotypes: *dor* ^+*/*+^*, dor* ^+*/CRISPR*^*,* and *dor *^*CRISPR/CRISPR*^*.* Non-lethal genotyping allowed the screening of G_2_ adults to isolate the *dor *^*CRISPR/CRISPR*^ genotype and subsequent set up of single pair crosses according to the nature of the induced mutation found by sequencing. G_3_ eggs from each single pair cross were collected three times, and adults were subjected to non-lethal genotyping to confirm the strain's stability. CRISPR strains with germline mutations of different natures were isolated and kept under laboratory conditions.

### In-situ hybridization

In-situ hybridization was performed as described in Gouvi et al. and Sollazzo et al. [18, 94]. Polytene chromosome preparations were prepared from third instar larvae salivary glands of *Ceratitis capitata* EgII strain, according to Mavragani-Tsipidou et al. 2014 [95]. The DNA labeling was performed using the “DIG-DNA Labeling and Detection” kit (Roche, Germany) following the manufacturer’s instructions. Well-spread nuclei were analyzed for the identification of the hybridization signals. Hybridization sites were photographed at a combined magnification of 60 × and 100 × using a phase contrast microscope DM2000 Led (Leica) and a camera DMC5400 (Leica). They were identified by using the salivary gland chromosome maps of *C. capitata* as reference [96].

### Temperature-sensitive lethal test

Temperature-sensitive lethal tests (TSLT) were performed on CRISPR strains (two generations after the establishment of homozygous strains) and control strains (EgII, SEIB, and *wp tsl*) as previously described [11, 17, 97] to assess their temperature sensitivity. Briefly, three replicates of 100 eggs each were prepared, for each of the seven temperatures tested, resulting in the collection of 2100 eggs in total on a daily basis. This egg collection scheme was repeated for three consecutive days. For each replicate, the eggs were placed on black strips on top of 90 × 15 mm Petri dishes filled with larval carrot diet and kept at 25 °C for 24 h. Each set of three replicates was incubated at different temperatures (25, 31, 32, 33, 34, 35, and 36 °C) for 24 h. After the heat shock, Petri dishes were placed at 25 °C to complete their development. 5-, 15-, and 23-days post egg collection, egg hatching, pupal recovery, and adult recovery rates were determined. Egg hatching, pupal recovery, and adult emergence rates were calculated for single replicate using the number of collected embryos (100) as a reference and the number of hatched eggs after five days, the number of puparia obtained after fifteen days, and the number of eclosed adults after twenty-three days, respectively.

### Statistical analysis

All statistical analyses were performed using R version 4.2.0 [98]. All datasets of this study represent recovery rates (egg hatching, pupal recovery, and adult emergence) and were, therefore, analyzed using a GLM-binomial family or a GLM-quasi-binomial family, when overdispersion was detected [99]. The DHARMA package was used to check if the simulated dispersion is equal to the observed dispersion and identify overdispersion in the generalized linear models (GLM) [100]. In the case of overdispersion, a GLM-quasibinomial model using a logit link function was employed to address it [101]. The chi-square test for the GLM-binomial models and an F-test for the GLM-quasi-binomial models were used to analyze deviance. The goodness-of-fit of the models was inspected with simulation envelopes of half-normal plots [102]. The ‘estimated marginal means’ (emmeans) package was used for the pairwise comparisons of the fitted model estimates [103]. For all data, the significance level was set to α = 0.05.

### Supplementary Information

**Additional file 1: Figure S1.** In situ hybridization of Ccdor in position 77B on the right arm of the C. capitata polytene chromosome 5. **Table S1.** SNPs detected in the coding region of deep orange gene in the C. capitata wp tsl mutant strain. The position of SNPs in the Ccdor CDS, nucleotide change, type of polymorphism, and the amino acid change when it occurs are shown. **Table S2.** List of primers and sgRNAs used in this study. **Figure S2.** Amino acid sequence alignment of different insect species’ deep orange homologs using MUSCLE. Identical residues are shaded in black, and conserved residues are shaded grey. Red dashes represent the six SNPs (that lead to amino acid changes) found in tsl mutant strains. **Figure S3.** Secondary structure and disorders prediction of CcDOR wild type protein done with the Phyre2 online tool (http://www.sbg.bio.ic.ac.uk/phyre2). **Table S3.** Summary of the microinjections performed using sgRNA_NHEJ and sgRNA_HDR + ssODN to induce Ccdor gene knock-out and introduce the E839K point mutation, respectively. **Figure S4.** CRISPR/Cas9 NHEJ induced mutation in G0. Sanger sequencing of seven dead pupae recovered from sgRNA_NHEJ injections targeting Ccdor gene exon 4. The red arrow indicates the Cas9 cutting site. Poor sequencing results upstream of the Cas9 cutting site could be due to mosaicism induced by CRISPR NHEJ. **Table S4.** Egg hatching, pupal recovery, and adult emergence rates (shown as mean and standard deviation) of Ceratitis capitata control and CRISPR strains at different temperatures. **Table S5.** Pairwise comparisons of egg hatching rates of Ceratitis capitata control and CRISPR strains at different temperatures. **Table S6.** Pairwise comparisons of egg hatching rates between Ceratitis capitata control and CRISPR strains egg at different temperatures. **Table S7.** Pairwise comparisons of pupal recovery rates of Ceratitis capitata control and CRISPR strains at different temperatures. **Table S8.** Pairwise comparisons of adult recovery rates of Ceratitis capitata control and CRISPR strains at different temperatures.

## Data Availability

The datasets generated and/or analyzed during the current study are available at NCBI under the accession numbers GCF_000347755.2 (*Ccap* 2.1), GCA_905071925 (*Ccap 3.2.1*), and the NCBI BioProject No. PRJEB57574.

## Abbreviations

AW-IPM: Area-Wide Integrated Pest Management
SIT: Sterile insect technique
GSS: Genetic sexing strain
FAO/IAEA: Food and Agriculture Organization/International Atomic Energy Agency
CRP: Coordinated research projects
wp: White pupae
tsl: Temperature sensitive lethal
EgII: Egypt II
SEIB: Seibersdorf
dor: Deep orange
Ccdor: Ceratitis capitata deep orange
Dmdor: Drosophila melanogaster deep orange
CRISPR: Clustered Regularly Interspaced Short Palindromic Repeats
NHEJ: Non-homologous end joining
HDR: Homology-Directed Repair
PAM: Protospacer adjacent motif
sgRNA: Single guide RNA
DSB: Double-stranded DNA break
ssODN: Single-stranded oligodeoxynucleotide
SNP: Single nucleotide polymorphism
NGS: Next-generation sequencing
RH: Relative humidity
PCR: Polymerase chain reaction
aa: Amino acid
bp: Base pair
G_x_: Generation
Del: Deletion
Dup: Duplication
TSLT: Temperature-sensitive lethal test
MVB: Multivesicular body
