# Supplementary material for: Deep orange gene editing triggers temperature-sensitive lethal phenotypes in Ceratitis capitata
Source: BMC Biotechnol. 2024 Feb 1;24:7. doi: 10.1186/s12896-024-00832-x (PMC10835909; doi:10.1186/s12896-024-00832-x)
Supplement: Supplementary file 1 — Additional file 1: Figure S1. In situ hybridization of Ccdor in position 77B on the right arm of the C. capitata polytene chromosome 5. Table S1. SNPs detected in the coding region of deep orange gene in the C. capitata wp tsl mutant strain. The position of SNPs in the Ccdor CDS, nucleotide change, type of polymorphism, and the amino acid change when it occurs are shown. Table S2. List of primers and sgRNAs used in this study. Figure S2. Amino acid sequence alignment of different insect species’ deep orange homologs using MUSCLE. Identical residues are shaded in black, and conserved residues are shaded grey. Red dashes represent the six SNPs (that lead to amino acid changes) found in tsl mutant strains. Figure S3. Secondary structure and disorders prediction of CcDOR wild type protein done with the Phyre2 online tool (http://www.sbg.bio.ic.ac.uk/phyre2). Table S3. Summary of the microinjections performed using sgRNA_NHEJ and sgRNA_HDR + ssODN to induce Ccdor gene knock-out and introduce the E839K point mutation, respectively. Figure S4. CRISPR/Cas9 NHEJ induced mutation in G0. Sanger sequencing of seven dead pupae recovered from sgRNA_NHEJ injections targeting Ccdor gene exon 4. The red arrow indicates the Cas9 cutting site. Poor sequencing results upstream of the Cas9 cutting site could be due to mosaicism induced by CRISPR NHEJ. Table S4. Egg hatching, pupal recovery, and adult emergence rates (shown as mean and standard deviation) of Ceratitis capitata control and CRISPR strains at different temperatures. Table S5. Pairwise comparisons of egg hatching rates of Ceratitis capitata control and CRISPR strains at different temperatures. Table S6. Pairwise comparisons of egg hatching rates between Ceratitis capitata control and CRISPR strains egg at different temperatures. Table S7. Pairwise comparisons of pupal recovery rates of Ceratitis capitata control and CRISPR strains at different temperatures. Table S8. Pairwise comparisons of adult recovery rates of Ceratitis [file 12896_2024_832_MOESM1_ESM.docx]

Supplementary Material

***Deep orange* gene editing triggers temperature sensitive lethal phenotypes in *Ceratitis capitata***

**Germano Sollazzo^1,2,^**^$^**, Katerina Nikolouli^1^, Georgia Gouvi^1,3,^**^$^**, Roswitha A. Aumann^2^, Marc F. Schetelig^2,*^, Kostas Bourtzis^1,*^**

^1^ Insect Pest Control Laboratory, Joint FAO/IAEA Centre of Nuclear Techniques in Food and Agriculture, Friedensstrasse 1, 2444 Seibersdorf, Austria.

^2^ Justus-Liebig-University Gießen, Institute for Insect Biotechnology, Department of Insect Biotechnology in Plant Protection, Winchesterstr. 2, 35394 Gießen, Germany.

^3^ Laboratory of Systems Microbiology and Applied Genomics, Department of Sustainable Agriculture, University of Patras, 2 G. Seferi St., 30100, Agrinio, Greece.

^$^ Present address: Department of Life Sciences, Imperial College London, Sir Alexander Fleming Building, South Kensington Campus, Imperial College Road, SW7 2AZ London, United Kingdom.

*Corresponding author:

Email: K.Bourtzis@iaea.org; Marc.Schetelig@agrar.uni-giessen.de


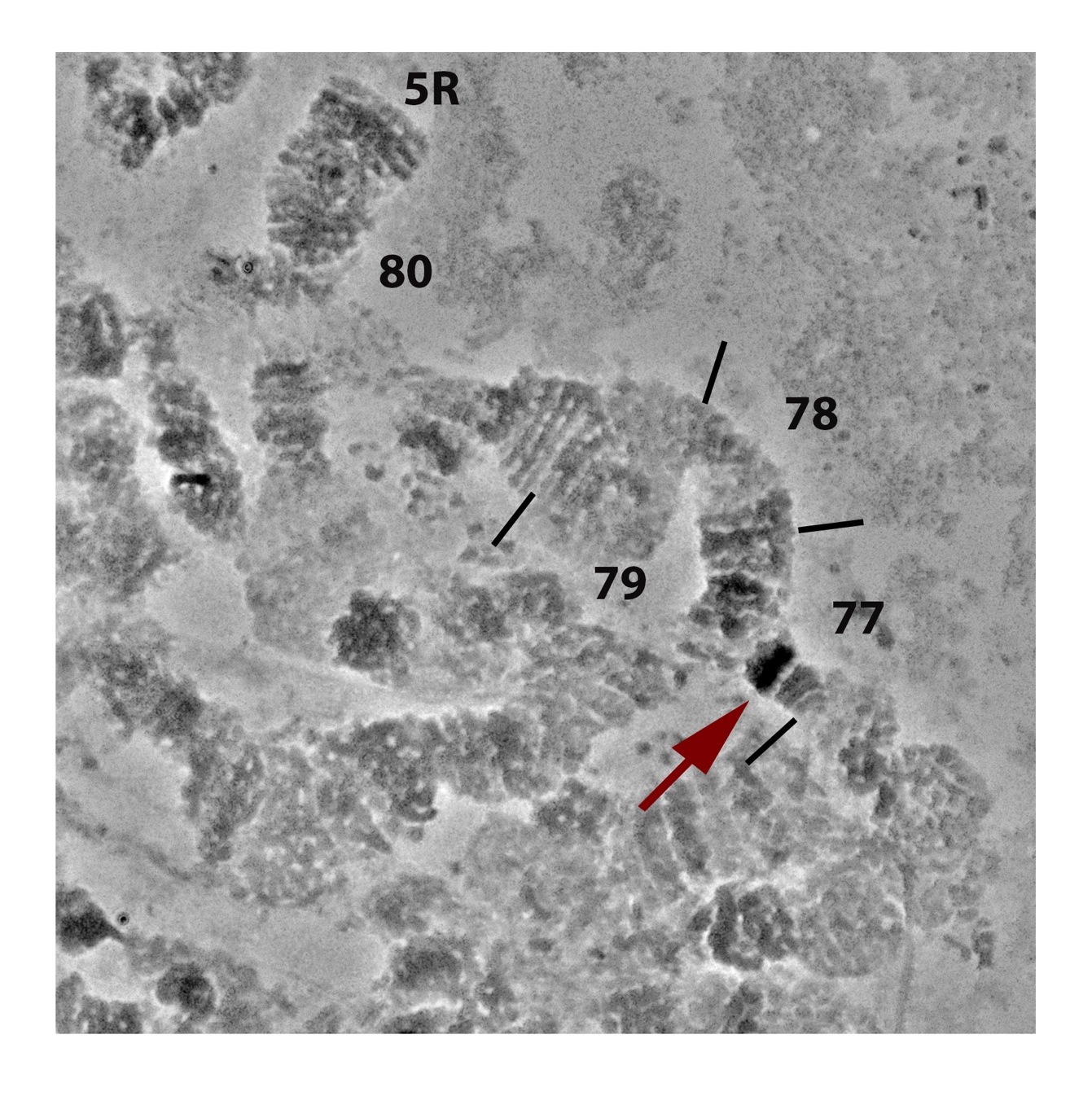


**Figure S1**. *In situ* hybridization of *Ccdor* in position 77B on the right arm of the *C. capitata* polytene chromosome 5.

**Table S1. SNPs detected in the coding region of *deep orange* gene in the *C. capitata* *wp tsl* mutant strain.** The position of SNPs in the *Ccdor* CDS, nucleotide change, type of polymorphism, and the amino acid change when it occurs are shown.

| Position in *Ccdor* gene (bp) | Position in *Cc*DOR protein (aa) | Nucleotide change | Polymorphism type | Amino acid change |
| --- | --- | --- | --- | --- |
| 60 |  | A -> T | SNP (transversion) |  |
| 87 |  | T -> C | SNP (transition) |  |
| 249 | 83 | A -> G | SNP (transition) | I -> M |
| 362 |  | T -> C | SNP (transition) |  |
| 367 | 100 | T -> G | SNP (transversion) | D -> E |
| 424 |  | C -> T | SNP (transition) |  |
| 625 |  | A -> G | SNP (transition) |  |
| 735 |  | C -> T | SNP (transition) |  |
| 781 |  | C -> T | SNP (transition) |  |
| 864 |  | G -> T | SNP (transversion) |  |
| 1,032 |  | A -> C | SNP (transversion) |  |
| 1,170 |  | C -> G | SNP (transversion) |  |
| 1,184 | 313 | A -> C | SNP (transversion) | H -> P |
| 1,188 |  | T -> C | SNP (transition) |  |
| 1,194 |  | A -> C | SNP (transversion) |  |
| 1,212 |  | C -> T | SNP (transition) |  |
| 1,227 |  | A -> G | SNP (transition) |  |
| 1,240 |  | T -> C | SNP (transition) |  |
| 1,431 |  | A -> T | SNP (transversion) |  |
| 1,455 |  | T -> C | SNP (transition) |  |
| 1,618 | 458 | A -> T | SNP (transversion) | I -> L |
| 1,650 |  | G -> A | SNP (transition) |  |
| 1,932 |  | A -> G | SNP (transition) |  |
| 2,016 |  | C -> T | SNP (transition) |  |
| 2,028 |  | T -> C | SNP (transition) |  |
| 2,070 |  | C -> T | SNP (transition) |  |
| 2,238 |  | T -> C | SNP (transition) |  |
| 2,346 |  | G -> T | SNP (transversion) |  |
| 2,529 |  | C -> T | SNP (transition) |  |
| 2,631 |  | G -> A | SNP (transition) |  |
| 2,766 |  | T -> C | SNP (transition) |  |
| 2,889 | 839 | G -> A | SNP (transition) | E -> K |
| 2,924 |  | A -> G | SNP (transition) |  |
| 3,131 |  | A -> G | SNP (transition) |  |
| 3,192 | 940 | C -> A | SNP (transversion) | L -> I |
| 3,248 |  | T -> G | SNP (transversion) |  |

**Table S2.** List of primers and sgRNAs used in this study

| Name | Sequence | Purpose |
| --- | --- | --- |
| P58_2kb_F | GTTTCCGCATAGAAATCGGCT | Sanger sequencing |
| P58_2kb_R | CACTGAACTTTCCAGCAGCG | Sanger sequencing |
| P58_3kb_F | TCCTGAATGAAAAGTTAACCATTTGC | Sanger sequencing |
| P58_3kb_R | GAAACTGAGTTAAACTATTCACTGT | Sanger sequencing |
| P58_F1 | GGTTCATAAATATCATCATTCC | Sanger sequencing |
| P58_F2 | AATAGTAACAGTCAGCCGCAA | Sanger sequencing |
| P58_F3 | TCAAAATCGGAGAAAAATGGCA | Sanger sequencing |
| P58_F4 | TATAGCATATTCCAAATAA | Sanger sequencing |
| P58_F5 | CCGTCGGGGATAATTGATTTGTG | Sanger sequencing |
| P58_F6 | TGTTCATGGAAATACTCCTCGT | Sanger sequencing |
| P58_F7 | TCTTCGCCGTGCATTTGTTG | Sanger sequencing |
| P58_F8 | TGCTGATAATCCACTGGTTTGTG | Sanger sequencing |
| P58_F9 | CGAACGACGACATTTTACTCGT | Sanger sequencing |
| P58_NHEJ_geno_F | TGTTCATGGAAATACTCCTCGT | No-lethal genotyping |
| P58_NHEJ_geno_R | TAACAAAGTCATCTTAGAAA | No-lethal genotyping |
| P58_HDR_geno_F | CGTTCACTTGATCCCAATCGC | No-lethal genotyping |
| P58_HDR_geno_R | TGCGGCTGTTGAATTAGCAC | No-lethal genotyping |
| P58_probe_F | CTAATTCAACAGCCGCAGCC | *In situ* hybridization |
| P58_probe_R | TGCACCTAACGTCCTCCTTG | *In situ* hybridization |
| sgRNA_NHEJ | TCAAAATGCACCACGTGCCATGG | CRISPR NHEJ |
| sgRNA_HDR | GGATGAATGTGATAAACAAGCGG | CRISPR HDR |
| ssODN_E839K_sense | AAAGATTACAATTTTCCTGTGCGCTAATGCGTATGCTTCTCTCACGCACATTTTGTAAATCTTTCATAACACGTTTGGCCTGTTTATCACATTCATCCATTTCGTGTTTTAATTCTTGAATTTTTTGATTGTAATCCTGAATGAAAAGGTG | CRISPR HDR |
| ssODN_E839K_anti | CACCTTTTCATTCAGGATTACAATCAAAAAATTCAAGAATTAAAACACGAAATGGATGAATGTGATAAACAGGCCAAACGTGTTATGAAAGATTTACAAAATGTGCGTGAGAGAAGCATACGCATTAGCGCACAGGAAAATTGTAATCTTT | CRISPR HDR |

**Figure S2.** **Amino acid sequence alignment of different insect species’ *deep orange* homologs using MUSCLE.** Identical residues are shaded in black, and conserved residues are shaded grey. Red dashes represent the six SNPs (that lead to amino acid changes) found in *tsl* mutant strains.

**Figure S3**. Secondary structure and disorders prediction of *CcDOR* wild type protein done with the Phyre^2^ online tool (<http://www.sbg.bio.ic.ac.uk/phyre2>).

**Table S3.** Summary of the microinjections performed using sgRNA_NHEJ and sgRNA_HDR + ssODN to induce *Ccdor* gene knock-out and introduce the E839K point mutation, respectively.

| Injection | sgRNA | ssODN | # embryos injected | # G_0_ pupae  (% survival) ^a^ | # G_0_ adults  (% survival) ^b^ | #G_0_ germline mutants ^c^ | Detected mutation | |
| --- | --- | --- | --- | --- | --- | --- | --- | --- |
|  |  |  |  |  |  |  | G_1_ mutant families | Mutation |
| NHEJ | sgRNA_NHEJ | no | 586 | 70 (11.9 %) | 55 (9.4 %) | 7 (12.7 %) ^d^ | 0 | mosaicism |
| HDR | sgRNA_HDR | ssODN_E839K_sense | 255 | 28 (11.0 %) | 28 (11.0 %) | 1 (4.5 %) | 1 | Knock-in E839K |
|  |  | ssODN_E839K_antisense | 100 | 4 (4.0 %) | 3 (3.0 %) | 3 (100.0 %) | 2 | Deletion (12 bp)  Knock-in E839K + Duplication (51 bp) |

^a^ Pupal recovery rate is presented in parentheses as the percentage of G_0_ pupae out of the total number of injected embryos.

^b^ Adult recovery rate is presented in parentheses as the percentage of G_0_ adults out of the total number of injected embryos.

^c^ Mutagenesis efficiency is presented in parentheses as the percentage of G_0_ individuals with a CRISPR-induced mutation out of the total number of G_0_ adults obtained.

^d^ All died at mid- or late-pupal stage.

**Figure S4. CRISPR/Cas9 NHEJ induced mutation in G_0_.** Sanger sequencing of seven dead pupae recovered from sgRNA_NHEJ injections targeting *Ccdor* gene exon 4. The red arrow indicates the Cas9 cutting site. Poor sequencing results upstream of the Cas9 cutting site could be due to mosaicism induced by CRISPR NHEJ.

**Table S4**: Egg hatching, pupal recovery, and adult emergence rates (shown as mean and standard deviation) of *Ceratitis capitata* control and CRISPR strains at different temperatures.

| **Strain** | **Temperature (°C)** | **Egg hatching (%)** | **Pupal recovery (%)** | **Adult emergence (%)** |
| --- | --- | --- | --- | --- |
| EgII | 25 | 98.00 ± 1.91 | 89.11 ± 6.75 | 89.11 ± 6.75 |
|  | 31 | 95.89 ± 0.96 | 87.67 ± 5.78 | 87.44 ± 5.75 |
|  | 32 | 97.22 ± 0.96 | 90.33 ± 6.14 | 91.11 ± 4.11 |
|  | 33 | 96.89 ± 1.47 | 87.00 ± 6.36 | 86.44 ± 7.27 |
|  | 34 | 96.56 ± 1.40 | 77.11 ± 6.46 | 84.67 ± 8.95 |
|  | 35 | 92.67 ± 2.81 | 64.89 ± 3.81 | 63.44 ± 4.63 |
|  | 36 | 78.00 ± 3.59 | 20.89 ± 13.10 | 19.00 ± 11.93 |
| SEIB | 25 | 96.56 ± 1.54 | 89.56 ± 4.52 | 89.56 ± 4.52 |
|  | 31 | 94.89 ± 1.28 | 86.22 ± 9.10 | 86.22 ± 9.10 |
|  | 32 | 95.56 ± 2.04 | 88.00 ± 4.24 | 88.00 ± 4.24 |
|  | 33 | 94.11 ± 2.94 | 78.00 ± 4.08 | 76.56 ± 4.13 |
|  | 34 | 92.89 ± 1.79 | 80.33 ± 4.42 | 77.78 ± 3.93 |
|  | 35 | 85.00 ± 2.81 | 56.89 ± 5.61 | 51.00 ± 6.35 |
|  | 36 | 62.33 ± 8.81 | 17.56 ± 11.07 | 16.00 ± 9.74 |
| *wp tsl* | 25 | 92.78 ± 1.93 | 81.00 ± 3.41 | 80.67 ± 3.41 |
|  | 31 | 79.00 ± 2.40 | 38.22 ± 4.12 | 29.11 ± 5.39 |
|  | 32 | 85.78 ± 2.37 | 52.11 ± 6.23 | 23.11 ± 3.42 |
|  | 33 | 9.11 ± 1.92 | 2.00 ± 0.58 | 0.33 |
|  | 34 | 0.00 | 0.00 | 0.00 |
|  | 35 | 0.00 | 0.00 | 0.00 |
|  | 36 | 0.00 | 0.00 | 0.00 |
| *dor E839K* | 25 | 95.67 ± 1.58 | 89.00 ± 5.30 | 87.56 ± 4.64 |
|  | 31 | 96.22 ± 0.38 | 88.00 ± 3.79 | 87.44 ± 3.99 |
|  | 32 | 94.56 ± 1.46 | 84.89 ± 5.09 | 84.11 ± 5.46 |
|  | 33 | 95.44 ± 2.59 | 87.89 ± 2.04 | 86.89 ± 2.10 |
|  | 34 | 93.56 ± 2.35 | 84.56 ± 5.37 | 83.11 ± 5.55 |
|  | 35 | 89.11 ± 2.19 | 51.22 ± 8.74 | 50.33 ± 9.11 |
|  | 36 | 41.44 ± 10.75 | 7.44 ± 4.27 | 6.89 ± 3.60 |
| *dor 12del* | 25 | 91.33 ± 2.63 | 78.33 ± 3.94 | 73.22 ± 3.87 |
|  | 31 | 91.00 ± 1.49 | 78.67 ± 6.16 | 78.11 ± 5.63 |
|  | 32 | 89.44 ± 1.46 | 77.56 ± 4.93 | 74.89 ± 5.12 |
|  | 33 | 89.22 ± 3.99 | 70.89 ± 8.33 | 67.22 ± 8.13 |
|  | 34 | 86.78 ± 4.14 | 51.00 ± 6.70 | 37.89 ± 5.13 |
|  | 35 | 74.56 ± 5.06 | 25.67 ± 7.10 | 19.56 ± 5.28 |
|  | 36 | 17.22 ± 11.70 | 0.33 ± 0.58 | 0.33 ± 0.58 |
| *dor 51dup* | 25 | 93.22 ± 2.52 | 81.44 ± 2.11 | 80.44 ± 2.41 |
|  | 31 | 92.56 ± 2.49 | 80.33 ± 5.58 | 80.11 ± 5.73 |
|  | 32 | 90.78 ± 2.78 | 79.22 ± 5.63 | 78.44 ± 5.72 |
|  | 33 | 90.89 ± 1.28 | 75.22 ± 4.13 | 74.11 ± 4.40 |
|  | 34 | 88.11 ± 7.54 | 52.56 ± 6.98 | 49.33 ± 6.89 |
|  | 35 | 57.78 ± 9.94 | 7.67 ± 6.24 | 4.44 ± 4.50 |
|  | 36 | 0.11 ± 0.19 | 0.00 | 0.00 |

**Table S5**: Pairwise comparisons of egg hatching rates of *Ceratitis capitata* control and CRISPR strains at different temperatures.

| **Strain** | **Temperature [°C] pairwise comparisons** | **Estimate** | **Standard Error** | **df** | **z ratio** | ***p* value** |
| --- | --- | --- | --- | --- | --- | --- |
| EgII | 25 - 31 | 0.7843 | 0.585 | Inf | 1.340 | 0.1804 |
|  | 31 - 32 | -0.5733 | 0.548 | Inf | -1.047 | 0.2951 |
|  | 32 – 33 | 0.1752 | 0.592 | Inf | 0.296 | 0.7671 |
|  | 33 – 34 | 0.1500 | 0.547 | Inf | 0.274 | 0.7840 |
|  | 34 – 35 | 0.9927 | 0.441 | Inf | 2.249 | 0.0245 |
|  | 35 - 36 | 13.690 | 0.275 | Inf | 4.971 | <.0001 |
| SEIB | 25 - 31 | 0.5371 | 0.472 | Inf | 1.137 | 0.2555 |
|  | 31 - 32 | -0.1839 | 0.429 | Inf | -0.429 | 0.6680 |
|  | 32 – 33 | 0.3653 | 0.413 | Inf | 0.883 | 0.3770 |
|  | 33 – 34 | 0.2361 | 0.359 | Inf | 0.657 | 0.5111 |
|  | 34 – 35 | 0.9167 | 0.292 | Inf | 3.139 | 0.0017 |
|  | 35 - 36 | 12.687 | 0.204 | Inf | 6.205 | <.0001 |
| *wp tsl* | 25 - 31 | 13.265 | 0.278 | Inf | 4.764 | <.0001 |
|  | 31 - 32 | -0.4952 | 0.223 | Inf | -2.223 | 0.0262 |
|  | 32 – 33 | 40.664 | 0.256 | Inf | 15.906 | <.0001 |
|  | 33 – 34 | sanp | - | - | sanp | sanp |
|  | 34 – 35 | sanp | - | - | sanp | sanp |
|  | 35 - 36 | sanp | - | - | sanp | sanp |
| *dor E839K* | 25 - 31 | -0.1881 | 0.475 | Inf | -0.396 | 0.6922 |
|  | 31 - 32 | 0.4873 | 0.448 | Inf | 1.087 | 0.2768 |
|  | 32 – 33 | -0.2324 | 0.418 | Inf | -0.556 | 0.5782 |
|  | 33 – 34 | 0.4459 | 0.401 | Inf | 1.111 | 0.2666 |
|  | 34 – 35 | 0.6450 | 0.319 | Inf | 2.024 | 0.0430 |
|  | 35 - 36 | 25.142 | 0.225 | Inf | 11.159 | <.0001 |
| *dor 12del* | 25 - 31 | 0.0462 | 0.303 | Inf | 0.152 | 0.8789 |
|  | 31 - 32 | 0.1947 | 0.289 | Inf | 0.674 | 0.5001 |
|  | 32 – 33 | 0.0254 | 0.276 | Inf | 0.092 | 0.9264 |
|  | 33 – 34 | 0.2506 | 0.262 | Inf | 0.958 | 0.3382 |
|  | 34 – 35 | 0.8431 | 0.221 | Inf | 3.815 | 0.0001 |
|  | 35 - 36 | 26.298 | 0.200 | Inf | 13.118 | <.0001 |
| *dor 51dup* | 25 - 31 | 0.1163 | 0.340 | Inf | 0.342 | 0.7326 |
|  | 31 - 32 | 0.2628 | 0.314 | Inf | 0.836 | 0.4033 |
|  | 32 – 33 | -0.0148 | 0.297 | Inf | -0.050 | 0.9603 |
|  | 33 – 34 | 0.3252 | 0.280 | Inf | 1.160 | 0.2462 |
|  | 34 – 35 | 17.474 | 0.219 | Inf | 7.989 | <.0001 |
|  | 35 - 36 | 48.434 | 0.562 | Inf | 8.625 | <.0001 |

* Inf = Infinite degrees of freedom. Z test used for pairwise comparison is not affected by the number of observations.

sanp = statistical analysis not possible due to values of 0 (zero) in the calculations.

**Table S6**: Pairwise comparisons of egg hatching rates between *Ceratitis capitata* control and CRISPR strains egg at different temperatures.

| **Strain** | **Temperature [°C] pairwise comparisons** | **Estimate** | **Standard Error** | **df** | **z ratio** | ***p* value** |
| --- | --- | --- | --- | --- | --- | --- |
| *dor E839K*  vs  EgII | 25 | -0.8556 | 0.579 | Inf | -1.477 | 0.1398 |
|  | 31 | 0.1168 | 0.482 | Inf | 0.242 | 0.8087 |
|  | 32 | -0.9438 | 0.518 | Inf | -1.824 | 0.0682 |
|  | 33 | -0.5362 | 0.507 | Inf | -1.057 | 0.2903 |
|  | 34 | -0.8320 | 0.451 | Inf | -1.845 | 0.0651 |
|  | 35 | -0.4843 | 0.305 | Inf | -1.587 | 0.1124 |
|  | 36 | -16.295 | 0.183 | Inf | -8.896 | <.0001 |
| *dor E839K*  vs  SEIB | 25 | -0.3193 | 0.492 | Inf | -0.649 | 0.5162 |
|  | 31 | 0.4059 | 0.455 | Inf | 0.892 | 0.3722 |
|  | 32 | -0.2653 | 0.422 | Inf | -0.629 | 0.5291 |
|  | 33 | 0.3324 | 0.410 | Inf | 0.811 | 0.4173 |
|  | 34 | 0.1226 | 0.350 | Inf | 0.351 | 0.7258 |
|  | 35 | 0.3943 | 0.254 | Inf | 1.550 | 0.1211 |
|  | 36 | -0.8511 | 0.167 | Inf | -5.101 | <.0001 |
| *dor E839K*  vs  *wp tsl* | 25 | 0.6545 | 0.400 | Inf | 1.638 | 0.1014 |
|  | 31 | 21.691 | 0.379 | Inf | 5.727 | <.0001 |
|  | 32 | 11.866 | 0.327 | Inf | 3.629 | 0.0003 |
|  | 33 | 54.854 | 0.365 | Inf | 15.034 | <.0001 |
|  | 34 | 74.497 | 0.632 | Inf | 11.791 | <.0001 |
|  | 35 | 68.048 | 0.610 | Inf | 11.157 | <.0001 |
|  | 36 | 42.906 | 0.590 | Inf | 7.269 | <.0001 |
| *dor 12del*  vs  EgII | 25 | -17.344 | 0.529 | Inf | -3.280 | 0.0010 |
|  | 31 | -0.9962 | 0.394 | Inf | -2.530 | 0.0114 |
|  | 32 | -17.643 | 0.478 | Inf | -3.693 | 0.0002 |
|  | 33 | -16.145 | 0.445 | Inf | -3.628 | 0.0003 |
|  | 34 | -17.151 | 0.412 | Inf | -4.161 | <.0001 |
|  | 35 | -15.655 | 0.272 | Inf | -5.762 | <.0001 |
|  | 36 | -28.263 | 0.205 | Inf | -13.754 | <.0001 |
| *dor 12del*  vs  SEIB | 25 | -11.981 | 0.431 | Inf | -2.779 | 0.0054 |
|  | 31 | -0.7071 | 0.359 | Inf | -1.968 | 0.0491 |
|  | 32 | -10.857 | 0.372 | Inf | -2.922 | 0.0035 |
|  | 33 | -0.7459 | 0.330 | Inf | -2.261 | 0.0238 |
|  | 34 | -0.7605 | 0.298 | Inf | -2.554 | 0.0107 |
|  | 35 | -0.6868 | 0.213 | Inf | -3.221 | 0.0013 |
|  | 36 | -20.479 | 0.191 | Inf | -10.717 | <.0001 |
| *dor 12del*  vs  *wp tsl* | 25 | -0.2243 | 0.322 | Inf | -0.697 | 0.4860 |
|  | 31 | 10.561 | 0.256 | Inf | 4.118 | <.0001 |
|  | 32 | 0.3662 | 0.259 | Inf | 1.412 | 0.1579 |
|  | 33 | 44.071 | 0.272 | Inf | 16.196 | <.0001 |
|  | 34 | 65.667 | 0.605 | Inf | 10.859 | <.0001 |
|  | 35 | 57.236 | 0.594 | Inf | 9.637 | <.0001 |
|  | 36 | 30.938 | 0.598 | Inf | 5.177 | <.0001 |
| *dor* *51dup*  vs  EgII | 25 | -14.313 | 0.542 | Inf | -2.641 | 0.0083 |
|  | 31 | -0.7632 | 0.406 | Inf | -1.879 | 0.0602 |
|  | 32 | -15.993 | 0.483 | Inf | -3.308 | 0.0009 |
|  | 33 | -14.093 | 0.453 | Inf | -3.114 | 0.0018 |
|  | 34 | -15.845 | 0.416 | Inf | -3.808 | 0.0001 |
|  | 35 | -23.391 | 0.264 | Inf | -8.869 | <.0001 |
|  | 36 | -58.136 | 0.567 | Inf | -10.250 | <.0001 |
| *dor* *51dup*  vs  SEIB | 25 | -0.8950 | 0.447 | Inf | -2.002 | 0.0453 |
|  | 31 | -0.4742 | 0.373 | Inf | -1.272 | 0.2035 |
|  | 32 | -0.9208 | 0.379 | Inf | -2.430 | 0.0151 |
|  | 33 | -0.5407 | 0.340 | Inf | -1.589 | 0.1120 |
|  | 34 | -0.6299 | 0.303 | Inf | -2.077 | 0.0378 |
|  | 35 | -14.605 | 0.203 | Inf | -7.194 | <.0001 |
|  | 36 | -50.352 | 0.562 | Inf | -8.958 | <.0001 |
| *dor 51dup*  vs  *wp tsl* | 25 | 0.0788 | 0.343 | Inf | 0.230 | 0.8182 |
|  | 31 | 12.890 | 0.275 | Inf | 4.685 | <.0001 |
|  | 32 | 0.5311 | 0.270 | Inf | 1.968 | 0.0491 |
|  | 33 | 46.123 | 0.285 | Inf | 16.211 | <.0001 |
|  | 34 | 66.973 | 0.607 | Inf | 11.025 | <.0001 |
|  | 35 | 49.499 | 0.590 | Inf | 8.385 | <.0001 |
|  | 36 | 0.1065 | 0.798 | Inf | 0.133 | 0.8938 |

**Table S7**: Pairwise comparisons of pupal recovery rates of *Ceratitis capitata* control and CRISPR strains at different temperatures

| **Strain** | **Temperature [°C] pairwise comparisons** | **Estimate** | **Standard Error** | **df** | **z ratio** | ***p* value** |
| --- | --- | --- | --- | --- | --- | --- |
| EgII | 25 - 31 | -0.06162 | 0.378 | Inf | -0.163 | 0.8705 |
|  | 31 - 32 | -0.45807 | 0.427 | Inf | -1.073 | 0.2834 |
|  | 32 – 33 | 0.62500 | 0.415 | Inf | 1.506 | 0.1321 |
|  | 33 – 34 | 0.04157 | 0.355 | Inf | 0.117 | 0.9068 |
|  | 34 – 35 | 130.976 | 0.301 | Inf | 4.349 | <.0001 |
|  | 35 - 36 | 185.422 | 0.255 | Inf | 7.272 | <.0001 |
| SEIB | 25 - 31 | 0.25150 | 0.395 | Inf | 0.636 | 0.5248 |
|  | 31 - 32 | -0.15761 | 0.388 | Inf | -0.406 | 0.6847 |
|  | 32 – 33 | 0.87785 | 0.349 | Inf | 2.517 | 0.0119 |
|  | 33 – 34 | -0.27882 | 0.305 | Inf | -0.915 | 0.3604 |
|  | 34 – 35 | 164.128 | 0.272 | Inf | 6.045 | <.0001 |
|  | 35 - 36 | sanp | - | - | sanp | sanp |
| *wp tsl* | 25 - 31 | 199.296 | 0.287 | Inf | 6.945 | <.0001 |
|  | 31 - 32 | -0.50159 | 0.235 | Inf | -2.131 | 0.0331 |
|  | 32 – 33 | 156.827 | 0.599 | Inf | 2.619 | 0.0088 |
|  | 33 – 34 | sanp | - | - | sanp | sanp |
|  | 34 – 35 | sanp | - | - | sanp | sanp |
|  | 35 - 36 | sanp | - | - | sanp | sanp |
| *dor E839K* | 25 - 31 | 0.22102 | 0.405 | Inf | 0.546 | 0.5850 |
|  | 31 - 32 | 0.19784 | 0.372 | Inf | 0.532 | 0.5946 |
|  | 32 – 33 | -0.28113 | 0.380 | Inf | -0.740 | 0.4590 |
|  | 33 – 34 | 0.21361 | 0.385 | Inf | 0.554 | 0.5793 |
|  | 34 – 35 | 193.867 | 0.307 | Inf | 6.324 | <.0001 |
|  | 35 - 36 | 242.078 | 0.407 | Inf | 5.944 | <.0001 |
| *dor 12del* | 25 - 31 | -0.05689 | 0.320 | Inf | -0.178 | 0.8587 |
|  | 31 - 32 | -0.02248 | 0.326 | Inf | -0.069 | 0.9450 |
|  | 32 – 33 | 0.52300 | 0.304 | Inf | 1.722 | 0.0850 |
|  | 33 – 34 | 0.99789 | 0.254 | Inf | 3.924 | 0.0001 |
|  | 34 – 35 | 0.99886 | 0.244 | Inf | 4.093 | <.0001 |
|  | 35 - 36 | 240.694 | 0.885 | Inf | 2.721 | 0.0065 |
| *dor 51dup* | 25 - 31 | 0.05078 | 0.326 | Inf | 0.156 | 0.8764 |
|  | 31 - 32 | -0.04216 | 0.328 | Inf | -0.128 | 0.8978 |
|  | 32 – 33 | 0.35618 | 0.313 | Inf | 1.137 | 0.2555 |
|  | 33 – 34 | 117.814 | 0.263 | Inf | 4.479 | <.0001 |
|  | 34 – 35 | 198.186 | 0.308 | Inf | 6.438 | <.0001 |
|  | 35 - 36 | sanp | - | - | sanp | sanp |

* Inf = Infinite degrees of freedom. Z test used for pairwise comparison is not affected by the number of observations.

sanp = statistical analysis not possible due to values of 0 (zero) in the calculations.

**Table S8**: Pairwise comparisons of adult recovery rates of *Ceratitis capitata* control and CRISPR strains at different temperatures.

| **Strain** | **Temperature [°C] pairwise comparisons** | **Estimate** | **Standard Error** | **df** | **z ratio** | ***p* value** |
| --- | --- | --- | --- | --- | --- | --- |
| EgII | 25 - 31 | 15.2199 | 1.752 | Inf | 0.009 | 0.9931 |
|  | 31 - 32 | 105.754 | 1.672 | Inf | 0.632 | 0.5271 |
|  | 32 – 33 | 0.20673 | 1.142 | Inf | 0.181 | 0.8563 |
|  | 33 – 34 | 248.619 | 0.814 | Inf | 3.053 | 0.0023 |
|  | 34 – 35 | -155.782 | 0.624 | Inf | -2.496 | 0.0126 |
|  | 35 - 36 | 147.399 | 0.774 | Inf | 1.905 | 0.0568 |
| SEIB | 25 - 31 | 0.2477 | Inf | 0.345 | 0.313 | 0.2477 |
|  | 31 - 32 | 0.2477 | Inf | 0.221 | 0.292 | 0.2477 |
|  | 32 – 33 | 0.1753 | Inf | 0.010 | 0.9922 | 0.1753 |
|  | 33 – 34 | 0.718 | Inf | 0.773 | 0.4396 | 0.718 |
|  | 34 – 35 | 0.525 | Inf | 2.394 | 0.0167 | 0.525 |
|  | 35 - 36 | 0.644 | Inf | -0.267 | 0.7896 | 0.644 |
| *wp tsl* | 25 - 31 | 432.731 | 1.210 | Inf | 3.575 | 0.0004 |
|  | 31 - 32 | 138.861 | 0.321 | Inf | 4.327 | <.0001 |
|  | 32 – 33 | sanp | - | - | sanp | sanp |
|  | 33 – 34 | sanp | - | - | sanp | sanp |
|  | 34 – 35 | sanp | - | - | sanp | sanp |
|  | 35 - 36 | sanp | - | - | sanp | sanp |
| *dor E839K* | 25 - 31 | -0.95424 | 1.081 | Inf | -0.883 | 0.3772 |
|  | 31 - 32 | 0.37534 | 1.201 | Inf | 0.312 | 0.7547 |
|  | 32 – 33 | 0.21882 | 1.035 | Inf | 0.211 | 0.8326 |
|  | 33 – 34 | 0.41218 | 0.892 | Inf | 0.462 | 0.6442 |
|  | 34 – 35 | 0.01600 | 0.926 | Inf | 0.017 | 0.9862 |
|  | 35 - 36 | 211.953 | 1.221 | Inf | 1.736 | 0.0825 |
| *dor 12del* | 25 - 31 | -228.384 | 0.969 | Inf | -2.357 | 0.0184 |
|  | 31 - 32 | 161.074 | 1.011 | Inf | 1.593 | 0.1111 |
|  | 32 – 33 | 0.42645 | 0.560 | Inf | 0.761 | 0.4465 |
|  | 33 – 34 | 184.752 | 0.426 | Inf | 4.340 | <.0001 |
|  | 34 – 35 | -0.10195 | 0.384 | Inf | -0.266 | 0.7906 |
|  | 35 - 36 | sanp | - | - | sanp | sanp |
| *dor 51dup* | 25 - 31 | -149.992 | 1.601 | Inf | -0.937 | 0.3490 |
|  | 31 - 32 | 127.379 | 1.643 | Inf | 0.776 | 0.4380 |
|  | 32 – 33 | 0.41350 | 1.013 | Inf | 0.408 | 0.6832 |
|  | 33 – 34 | 147.168 | 0.760 | Inf | 1.936 | 0.0528 |
|  | 34 – 35 | 291.085 | 0.587 | Inf | 4.956 | <.0001 |
|  | 35 - 36 | sanp | - | - | sanp | sanp |

* Inf = Infinite degrees of freedom. Z test used for pairwise comparison is not affected by the number of observations.

sanp = statistical analysis not possible due to values of 0 (zero) in the calculations.
